# Turning Immersive Viewers into Analytical Workspaces: ASCRIBE-XR and Agent-Driven Scientific Visualization

**DOI:** 10.3390/jimaging12080393

**Published:** 2026-08-20

**Authors:** Ronald Pandolfi, Luke Weidner, James Sethian, Jeffrey Donatelli, Daniela Ushizima

**Affiliations:** 1 Applied Math and Computational Research Division, Lawrence Berkeley National Laboratory, Berkeley, CA 94720, USA; 2 Computer Science Department, Sonoma State University, Rohnert Park, CA 94928, USA; 3 Berkeley Institute for Data Science, University of California Berkeley, Berkeley, CA 94709, USA; 4 Department of Mathematics, University of California Berkeley, Berkeley, CA 94709, USA; 5 Bakar Computational Health Sciences Institute, University of California San Francisco, San Francisco, CA 94143, USA

**Keywords:** virtual reality, computer vision, agentic analysis, AI auditing, LLM

## Abstract

Scientific visualization is changing from passive observation to active, AI-assisted collaboration. While Extended Reality (XR) has proven valuable for comprehending dense 3D arrays, traditional VR applications are typically deployed in rigid, single-purpose, and monolithic architectures. In this paper, we present the evolution of ASCRIBE-XR: a virtual reality platform backed by remote computation that has been re-engineered into a dynamic, service-oriented ecosystem. We introduce three core innovations that make immersive data analysis easier, faster, and more flexible when using multimodal scientific imaging. First, a lightweight Python REST interface decouples XR logic from the rendering engine, enabling real-time, programmable scene customization and on-demand data generation. Second, we present a Specimen Catalog architecture that lets the platform pivot between radically different disciplines, ranging from archaeological heterogeneous concrete and fuel-cell membranes to the root system of a bioenergy grass, by describing each dataset through portable metadata rather than hard-coded application logic. Finally, we introduce a prompt-driven layer powered by the Claude Agent SDK, allowing researchers to generate, segment, and manipulate volumetric and mesh data through natural language dialogue within the virtual space. For example, applying foundation models such as the Segment Anything Model (SAM) to perform zero-shot segmentation on demand. By bridging human intent with remote computation, ASCRIBE-XR relaxes the constraints of conventional visualization tools, offering a highly adaptable, conversational platform for scientific discovery with human auditing.

## 1. Introduction

The adoption of virtual reality (VR) for scientific data exploration [1,2] has been accelerated by advancements in XR software libraries, consumer hardware, and game engines [3]. Immersive VR environments provide scientists with enhanced clarity and natural comprehension of complex spatial contexts compared to traditional 2D monitors [4]. Previously, we introduced ASCRIBE-XR to address the lack of free, open-source computational platforms designed for the comprehensive visualization of raw volumetric and mesh data [5,6]. Built using Godot and PC-VR technologies, the pre-released ASCRIBE-XR leveraged WebRTC and MQTT for multi-user synchronization and presented researchers with a menu of preloaded specimens to inspect, while the analysis that produced those specimens remained in external pipelines.

**Figure 1:**
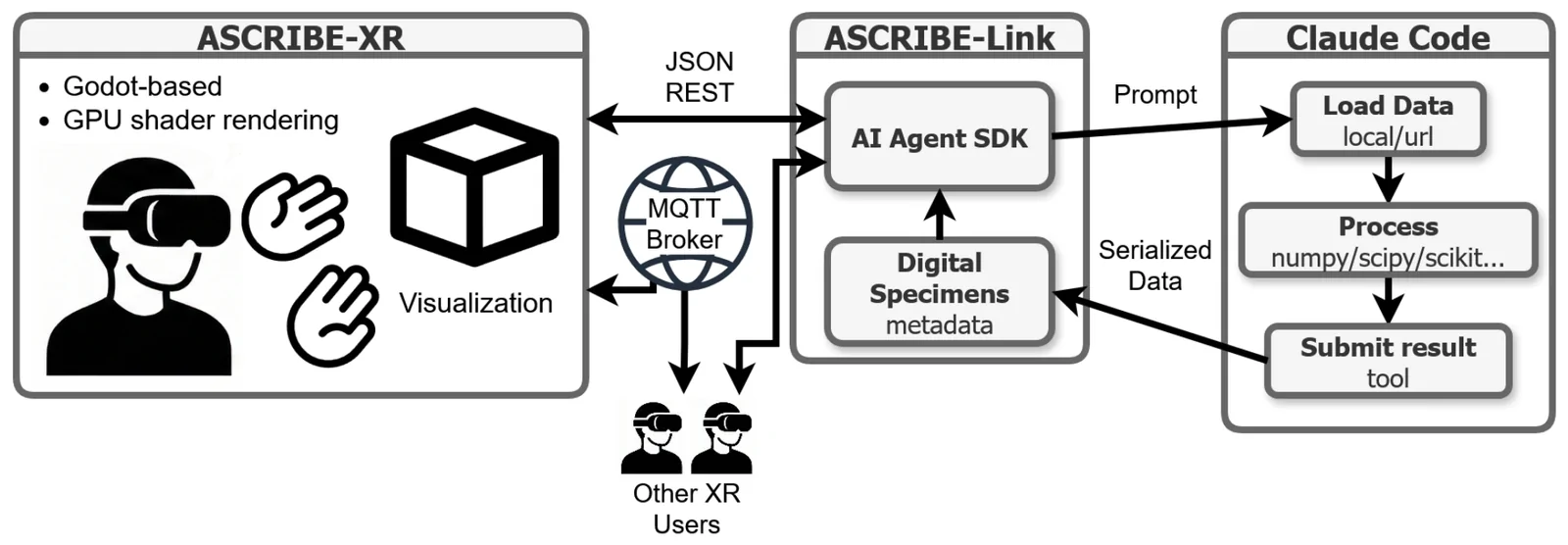
Service-oriented architecture of ASCRIBE-XR. The Godot-based VR client (left) performs GPU shader-based volume and mesh rendering and immersive interaction, and synchronizes with peer clients through an MQTT broker for multi-user sessions. It communicates with the ASCRIBE-Link server (center) over a JSON REST interface. ASCRIBE-Link hosts the Specimen Catalog (portable metadata describing each digital specimen) from whose per-function JSON Schemas the client procedurally generates the in-VR parameter controls, so that a newly registered specimen contributes its own interface without any client-side code. ASCRIBE-Link also hosts the optional Claude Agent SDK layer: a natural-language prompt issued from within the VR space is forwarded to a Claude agent running on the server (right), which can load data from local paths or URLs, author and execute analysis code against scientific Python libraries (NumPy, SciPy, scikit-image, and others), and return the product through a submit-result tool. The serialized mesh or volume is streamed back to the client and cached for the room, so that only the reduced result crosses the network, whether a decimated mesh or a subsampled volume, while the full-resolution source data remain resident on the server.

However, a persistent challenge in the development of virtual reality tools is the reliance on rigid, tightly coupled system architectures. To accommodate diverse workflows across scientific, industrial, and educational domains, XR tools must be flexible and easily extensible. In this paper, we describe the transformation of ASCRIBE-XR into a modular, agent-assisted platform capable of supporting prompt-driven data generation and cross-domain dataset reuse. Figure 1 summarizes the resulting service-oriented architecture. We present a theoretical comparison between ASCRIBE-XR and several recent VR tools in Section 3.

## 2. The ASCRIBE-XR Platform

Before describing the innovations introduced here, we summarize the base platform on which they build [5]. ASCRIBE-XR is written in the Godot game engine, an open-source engine chosen for its permissive licensing, its extensive community plugin ecosystem, and its native support for OpenXR, which is the vendor-neutral standard that Godot uses as its core interface to VR, AR, and mixed-reality hardware. Building on OpenXR allows a single client to run across a range of headsets: the application can execute standalone on the headset or, for the large volumetric datasets typical of synchrotron imaging, be driven from a workstation over PC-VR through a link layer such as Meta Link, SteamVR, or Virtual Desktop. Passthrough, which blends the physical laboratory with the virtual scene, is currently available on Meta Quest devices through a vendor-specific plugin. Rather than reimplementing low-level XR functionality, the client composes a set of community plugins with well-separated responsibilities: godot-xr-tools and godot_openxr_vendors for device interaction and controller tooling, and a player-networking plugin for multi-user sessions. The full dependency set is listed in Table A1.

The scientific value of the platform rests on its volumetric renderer. Dense 3D arrays, the native output of many imaging instruments, for example, tomographic data, are difficult to inspect on a flat display, where occlusion and the loss of depth cues obscure interior structure. ASCRIBE-XR renders volumes with a custom ray-marching shader, developed to replace the layer-stacking approach of the volume-rendering plugin, to obtain higher image quality at lower memory cost. The shader exposes the controls a scientist needs to interrogate a volume interactively; an adjustable look-up table (LUT) for color mapping, controllable opacity for revealing or suppressing density ranges, and exclusion planes that slice the volume so its interior can be examined directly. Mipmaps are generated for each 3D texture on load, improving both rendering quality at a distance and overall performance. Meshes and volumes are loaded dynamically at runtime, and once a specimen is present it can be freely scaled, recolored, and manipulated in the hand through the motion controllers. We detail the core architectural innovations in Section 4.

Beyond providing tools for single-user analysis, ASCRIBE-XR was designed for collaboration, allowing several researchers to share and inspect the same data in real time. We expect this to be particularly valuable for expert-to-novice knowledge transfer, where an experienced user can demonstrate an interpretation “in world,” though such collaborative tasks remain a motivating hypothesis rather than a tested result, as discussed in Section 5. Conventional multiplayer architectures depend on a statically provisioned server, whose setup and credential management are prohibitive for small research projects. ASCRIBE-XR instead links peers directly using WebRTC, so that once a session is established, the audio and data channels flow between clients rather than through an application server. Reaching that point still requires shared infrastructure, and we should be precise about what the design does and does not avoid. The lightweight MQTT protocol, originally designed for IoT telemetry, carries signaling through a broker that peers use to discover one another and exchange connection state, and traversing consumer NATs relies on a public STUN server, with a TURN relay required for the minority of networks that defeat a direct connection. What is avoided is a statically provisioned application server that must be sized, secured, and credentialed to carry the session data itself; the signaling broker is a lightweight, stateless, and shareable dependency by comparison. Section 6 records the defaults we distribute. Voice communication is carried over the same sessions using VOIP with Opus compression. Together these choices make collaborative sessions low-maintenance enough to be convened for casual feedback, not only for formally hosted demonstrations.

In its first release, ASCRIBE-XR [6] demonstrated this capability across markedly different imaging modalities: the interlocking mineral phases and porous networks of archaeological Roman concrete [7] imaged by X-ray tomography at the ALS, the fiber beds of ceramic matrix composites reconstructed from large microCT volumes, and the icosahedral capsid of the PBCV-1 virus recovered from fluctuation X-ray scattering [8]. In each case, 3D data segmented into binary images were reduced to surface meshes or rendered directly as volumes for immersive inspection. These applications established the platform’s utility, but they also exposed the limitation that motivates the present work: each new dataset and workflow had to be prepared outside the tool and loaded as a fixed specimen, with the analysis logic living in bespoke external pipelines rather than in the immersive environment itself.

## 3. Related Work: Server-Backed, Agentic Scientific Visualization

ASCRIBE-XR sits at the intersection of four lines of work that have largely developed independently: XR-capable volumetric visualization, client–server architectures for remote scientific computation, shared multi-user 3D analysis, and the integration of language models or agents with visualization software. We therefore compare against systems drawn from all four, rather than against the consumer and educational VR titles surveyed in our earlier report on the base platform [5]. Immersive applications built for formal education [3] or behavioral research [9] share ASCRIBE-XR’s display technology but not its purpose: they expose no programmable analysis surface, so a feature-by-feature comparison with them cannot speak to the originality of ASCRIBE-XR as a scientific platform. Therefore, they are excluded from this study and from Table 1.

### 3.1. Selection Criteria

To ensure a rigorous comparison, the VR platforms evaluated in Table 1 were selected based on the following inclusion criteria. Specifically, each platform must satisfy all of the following:

a. Scientific 3D data. The system supports interactive inspection or analysis of three-dimensional scientific data, that is, dense volumetric fields, surface meshes, or point sets, rather than abstract or tabular data. This excludes immersive analytics toolkits for abstract data such as IATK [10] and the natural-language-to-chart systems discussed below.
b. Overlap on at least one architectural axis. The system shares at least one of ASCRIBE-XR’s four defining characteristics: an immersive XR display mode, a client–server split placing computation off the display host, a shared multi-user analysis session, or an integrated language model or agent that drives visualization or analysis.
c. Verifiable documentation. Capabilities are documented in a peer-reviewed publication, an archival preprint, or maintained public developer documentation, so that each table entry can be checked against a citable source. Where a capability is asserted only by a vendor and we could not confirm it independently, it is marked “P” rather than “Y”.
d. Availability. The system is obtainable and maintained at the time of writing, whether as open source, free academic software, or a commercial product.

Within these criteria we sought breadth rather than exhaustiveness, covering general-purpose scientific visualization frameworks that have acquired an XR mode, domain-specific immersive analysis platforms, and the recent generation of agentic visualization systems. Two adjacent bodies of work are discussed but deliberately not tabulated: natural-language-to-chart systems such as LIDA [11], LLM4Vis [12], Data Formulator [13], and ChartGPT [14], which operate on tabular data and fail Criterion a; and agentic scientific-computing frameworks such as Coscientist [15], ChemCrow [16], and the AI Scientist [17], which execute generated code but are not visualization systems and fail Criterion b. Both establish that generated code execution is now a practical building block; neither places it inside an interactive visual session.

### 3.2. XR-Capable Volumetric Visualization and Client–Server Systems

General-purpose scientific visualization frameworks have added immersive front-ends to mature client–server back-ends. ParaView [18] is the reference case: its pvserver architecture has long supported MPI-parallel remote and in situ rendering, and the XRInterface plugin [19], with the immersive workflows built on it [20], brings that pipeline into OpenVR and OpenXR headsets and CAVE installations. Inviwo [21] likewise offers a general volumetric dataflow framework with C++ and Python interfaces and an OpenVR module. Both are extensible through scripting and plugins, but neither exposes a REST control plane usable from an arbitrary client, and neither provides an agent that can compose new analysis at run time.

Domain-specific platforms trade generality for depth. SlicerVR extends 3D Slicer into immersive medical intervention planning with collaborative sessions, local and remote [22,23]. UCSF ChimeraX [24] supports VR inspection of molecular structures and density maps [25] and a hub-and-spoke meeting mode for shared sessions. In microscopy, syGlass [26] provides out-of-core multiresolution rendering of very large image stacks, ConfocalVR and its expansion-microscopy successor [27,28] target confocal volumes, and DIVA [29] adds quantitative measurement inside 3D images. In astronomy, iDaVIE-v [30] renders FITS spectral cubes in VR. Nanome [31] remains the closest point of comparison among openly extensible platforms, combining a plugin API (Nanome Stacks), shared spatial workspaces, and a conversational assistant, but it is specialized for molecular structures and its analysis surface is bounded by that plugin API rather than open-ended code execution. Across this group, rendering is typically local to the workstation and the network carries scene state rather than computation, which is the inverse of the split ASCRIBE-XR adopts.

### 3.3. Language Models and Agents in Visualization

A fast-moving line of work that couples language models to scientific visualization is ChatVis [32,33], which generates ParaView Python scripts from natural language, executes them, and feeds errors back for repair; HiLSVA [34] adds human-in-the-loop capabilities, and SASAV [35] exclude those, while SciVisAgentBench [36] and related position work [37] attempt to standardize how agents are evaluated. These systems operate on a 2D desktop or in batch: the agent produces a rendering, but no one is inside the scene while it does so. On the immersive side, LLM-driven visualization agents have appeared outside the sciences, most directly in immersive binary reverse engineering [38], where an agent queries analysis tools and generates 3D views in VR, and language models have been used to analyze XR session recordings after the fact [39]. We are not aware of a published system that combines an immersive volumetric client, server-side execution of agent-authored analysis code against general scientific Python libraries, and delivery of the result into a shared multi-user scene.

### 3.4. Research Gap and Positioning

Table 1 shows that each of ASCRIBE-XR’s individual capabilities has precedent, but that they are distributed across systems rather than combined. Remote compute is mature in ParaView and absent from most immersive microscopy tools; multi-user sessions exist in SlicerVR, ChimeraX, and Nanome, but they synchronize scene state rather than share server-side computation; programmatic extensibility, where present, takes the form of in-process scripting or a plugin API rather than a language-neutral service interface; and in-session agents that author and execute analysis code appear only outside immersive scientific settings. Existing XR tools for scientific visualization are typically either proprietary or require substantial in-house development to achieve dynamic data loading and collaborative analysis, and many operate as closed, monolithic systems in which integrating a new imaging modality or analytics pipeline entails re-engineering the rendering path [26,31]. This coupling of data, analysis, and presentation is the constraint that ASCRIBE-XR’s service-oriented architecture is designed to overcome.

ASCRIBE-XR’s modularity is a direct consequence of this architectural separation. Rather than being hardcoded for a single predefined use-case, it is designed as an extensible ecosystem: through the Specimen Catalog, researchers register new 3D arrays, mesh files, and domain-specific metadata from which in-VR environments and controls are procedurally generated. This has allowed the platform to absorb new modules, such as vision–language tools [40] and plugins for CAD models, without re-engineering the core architecture.

**Table 1:** Comparison of ASCRIBE-XR with representative systems for interactive 3D scientific visualization and analysis, selected by the criteria in Section 3.1. Systems are grouped by the axis on which they are closest to ASCRIBE-XR. Entries describe each system’s design as documented by its developers or associated publications; they are not a ranking. Y = documented and generally available; P = partial, limited in scope, experimental, or documented only by the vendor; N = not documented. “Remote compute” denotes a client–server split in which analysis or rendering runs off the display host; “In-session agent” denotes a language model or agent that acts on the live scene or data during an interactive session.

System	Domain Scope/Primary Data	Programmatic Extensibility	XR Display	Multi-User	Remote Compute	In-Session Agent
*This work *						
ASCRIBE-XR [5,6]	Domain-agnostic; dense 3D arrays and meshes (microCT, tomography, FXS)	Open Python REST API; metadata-driven Specimen Catalog auto-generates in-VR controls	Y	Y	Y	Y
*General-purpose scientific visualization with an XR mode*						
ParaView + XRInterface [18,19,20]	Domain-agnostic; meshes, unstructured grids, volumes, particles	Python scripting (paraview.simple), C++ plugin API; no REST control plane	Y	P	Y	N
Inviwo [21]	Domain-agnostic; volumetric and dataflow scientific visualization	C++ modules and Python (inviwopy); OpenVR module	P	N	N	N
*Domain-specific immersive analysis platforms*						
3D Slicer + SlicerVR [22,23]	Medical imaging; DICOM, NIfTI, segmentations	Python scripting and C++/Python extension modules	Y	Y	P	N
UCSF ChimeraX [24,25]	Molecular structures, cryo-EM and microscopy density maps	Python command/bundle API and toolshed; remote-control interface	Y	Y	N	N
Nanome [31]	Molecular structures (PDB, SDF); drug discovery	Plugin API (Nanome Stacks)	Y	Y	P	P
syGlass [26]	Volumetric microscopy and neuroscience; TIFF/HDF5 stacks	Scripting advertised; no public plugin or REST API	Y	P	N	N
ConfocalVR/ ExMicroVR [27,28]	Confocal and expansion microscopy; multi-channel TIFF	None documented	Y	P	N	N
DIVA [29]	Microscopy volumes; native TIFF, quantitative measurement	None documented	Y	N	N	N
iDaVIE-v [30]	Radio astronomy; FITS spectral data cubes	Open source, but no scripting or plugin API	Y	N	N	N
*Language-model and agent-driven visualization*						
ChatVis [32,33]	Domain-agnostic scientific visualization via generated ParaView scripts	Agent authors and executes Python; batch, no interactive session	N	N	P	N
HiLSVA [34], SASAV [35]	Scientific visualization; human-in-the-loop and autonomous agents	Agent authors and executes visualization code on a 2D desktop	N	N	P	P
LLM agent for immersive binary RE [38]	Binary reverse engineering (non-scientific); program artifacts	Agent calls analysis tools and generates 3D views in VR	Y	N	Y	Y

Additionally, by keeping full-resolution datasets resident on remote compute servers and streaming only the reduced result, whether a decimated mesh or a downsampled volume, to the client, it targets the tension between the fidelity scientists need and the limits of consumer-grade XR hardware, and it does so with an architecture intended to reduce latency-related discomfort during interaction [41,42]. The contribution of the present work is therefore not a single new capability but the specific combination of a server-side compute, a metadata-driven catalog that generates its own in-VR controls, and a code-authoring agent, all assembled into an open-source, domain-agnostic platform.

## 4. Core Architectural Innovations

ASCRIBE-XR’s transition to a customizable platform is driven by three primary innovations:

### 4.1. Python REST Interface

To move beyond monolithic constraints, ASCRIBE-XR now integrates a Python REST interface that allows developers to build and extend XR applications via lightweight APIs. The server is implemented with the Litestar ASGI framework and exposes endpoints for browsing the Specimen Catalog, querying the parameter schema of each registered processing function, and invoking those functions with JSON-encoded arguments; the Godot client communicates with it over HTTP. A complementary WebSocket channel supports an optional federation mode, in which remote worker nodes register their specimens with a relay server so that a single client sees an aggregated catalog.

This decoupling of XR logic from the rendering engine provides:

- Customizable Endpoints: Programmatic control over XR scenes and on-demand generation of meshes and volumes.
- Multiplayer Result Caching: Room-scoped caching of computed results, so collaborators who request the same computation receive a cached result rather than triggering redundant work.
- Pipeline Integration: Simplified connection to existing analytics, computer vision, or data visualization pipelines written in Python.

### 4.2. Specimen Catalog for Multi-Domain Reuse

Our previous implementation allowed users to manually load specific file formats, such as OBJ, STL, and FBX meshes, raw binary volumes, or zipped image stacks. To scale data management, we introduce the Specimen Catalog architecture. Each specimen is described by a portable JSON metadata record that declares its display name, data type (mesh, volume, point cloud, or image), thumbnail, and tags, and, for procedurally generated specimens, the processing function that produces it together with a JSON Schema describing that function’s parameters. The catalog scans a specimens directory, indexes these records, and presents them to the XR client as a unified, browsable collection.

Because a new discipline is added by authoring metadata rather than modifying application logic, the same platform can host radically different datasets side by side. This metadata-driven design allows ASCRIBE-XR to:

- Support the cross-domain reuse of datasets and the parameter UI generated automatically from their schemas.
- Add new specimens, whether static files or parametric generators, without rebuilding the client.
- Aggregate specimens from multiple hosts through the federation relay, providing a scalable backbone for distributed collaborative environments.

Rather than hard-coding a single use-case, the Specimen Catalog acts as a generic description layer: the JSON Schema attached to each dynamic specimen is translated automatically into interactive controls within the VR environment, so adapting the system to a new domain is a matter of registering data and functions, not re-engineering the platform.

### 4.3. Claude Agent SDK for Prompt-Based Data Generation

Bridging human intent and computational execution, ASCRIBE-XR integrates the Claude Agent SDK as an optional prompt-driven generation layer, enabled at startup with the --enable-agent flag. When active, the server registers an ai_generate function that forwards a natural-language prompt to a Claude agent, allowing users to request data directly from within the VR space.

Beyond conversational reasoning, the agent operates with a general-purpose tool surface, with file Read/Write and Bash execution on the server, optionally wrapped in a Firejail confinement profile on Linux hosts (Section 6), augmented by an in-process MCP (Model Context Protocol) server that exposes four tools for returning results to the platform:

- submit_mesh: Returns the vertices, indices, and normals of a generated mesh.
- submit_mesh_file: Returns a path to a mesh serialized on disk, for geometry too large to pass inline.
- submit_volume: Returns a volumetric array together with its shape, data type, and spacing.
- submit_volume_file: Returns a path to a volume serialized on disk, for data too large to pass inline. The agent’s system prompt is generally encouraged to use this route.

Because the agent can author and execute code on the server, it is not limited to predefined parametric generators: it can install and invoke external libraries to carry out an analysis described in natural language and return the product to the shared scene. A researcher can thus request “a DNA double helix with ten base pairs” and receive a generated mesh, or apply a promptable foundation model such as the Segment Anything Model (SAM) [43,44] to segment a structure of interest zero-shot, with the resulting geometry streamed back into VR.

## 5. Results

In earlier studies, ASCRIBE-XR demonstrated high efficacy in visualizing the complex interlocking mineral structures and porous networks of ancient Roman concrete using microCT data. With the new modular architecture, these structural analyses evolve into dynamic conversations.

For instance, a researcher exploring archaeological concrete can ask the agent in natural language to “extract the pore network as a surface.” The agent invokes a processing function over the REST interface; on the server, that function calculates threshold [45] for the microCT volume, extracts an isosurface with marching cubes, and smooths and decimates the result (using PyVista) before returning a lightweight mesh. The mesh is streamed back into the VR environment and cached for the room, so other participants in the same collaborative session receive it without recomputation.

A more capable agentic workflow is zero-shot segmentation. Rather than relying on a hand-tuned, dataset-specific pipeline, a researcher can ask the agent in natural language to “segment the dense phase in this volume.” The agent applies a promptable foundation model, such as the SAM [43] within its server-side working directory, requiring no task-specific training, and returns the segmented region as a labeled volume or surface mesh for inspection in VR. A complete worked instance of this pathway, which includes from a single natural-language prompt to a decimated mesh streamed back into the shared scene, is given for a plant root specimen in Section 5.2. All prompts utilized in this research work are included in Table A2 in the appendix to improve reproducibility.

### 5.1. Use-Case I: Porous Membranes for Fuel Cells

Gas diffusion layers (GDLs) are fibrous porous media that manage reactant and water transport in proton-exchange-membrane fuel cells, and their performance depends sensitively on the connectivity of the carbon-fiber network and the distribution of hydrophobic polytetrafluoroethylene (PTFE) binder. The high-resolution X-ray micro-CT dataset used here (acquired on a Thermo Scientific HeliScan scanner, by Thermo Fisher, Waltham, MA, USA) resolves the microstructural arrangement of carbon fibers, water, and air within GDLs treated with varying PTFE concentrations. Analyzing such volumes at equilibrium, after fluid flow has ceased and water has fully spanned the layer, allows the local contact angle and meniscus curvature to be quantified, and, combined with a pore-network model, links microscale wettability to macroscopic fuel-cell performance. Characterizing this microstructure normally calls for a segmentation pipeline tuned to the specimen, which in prior practice has motivated dedicated deep learning models such as 2D and 3D U-Nets for multiphase segmentation of unfiltered GDL tomograms [46,47].

**Figure 2:**
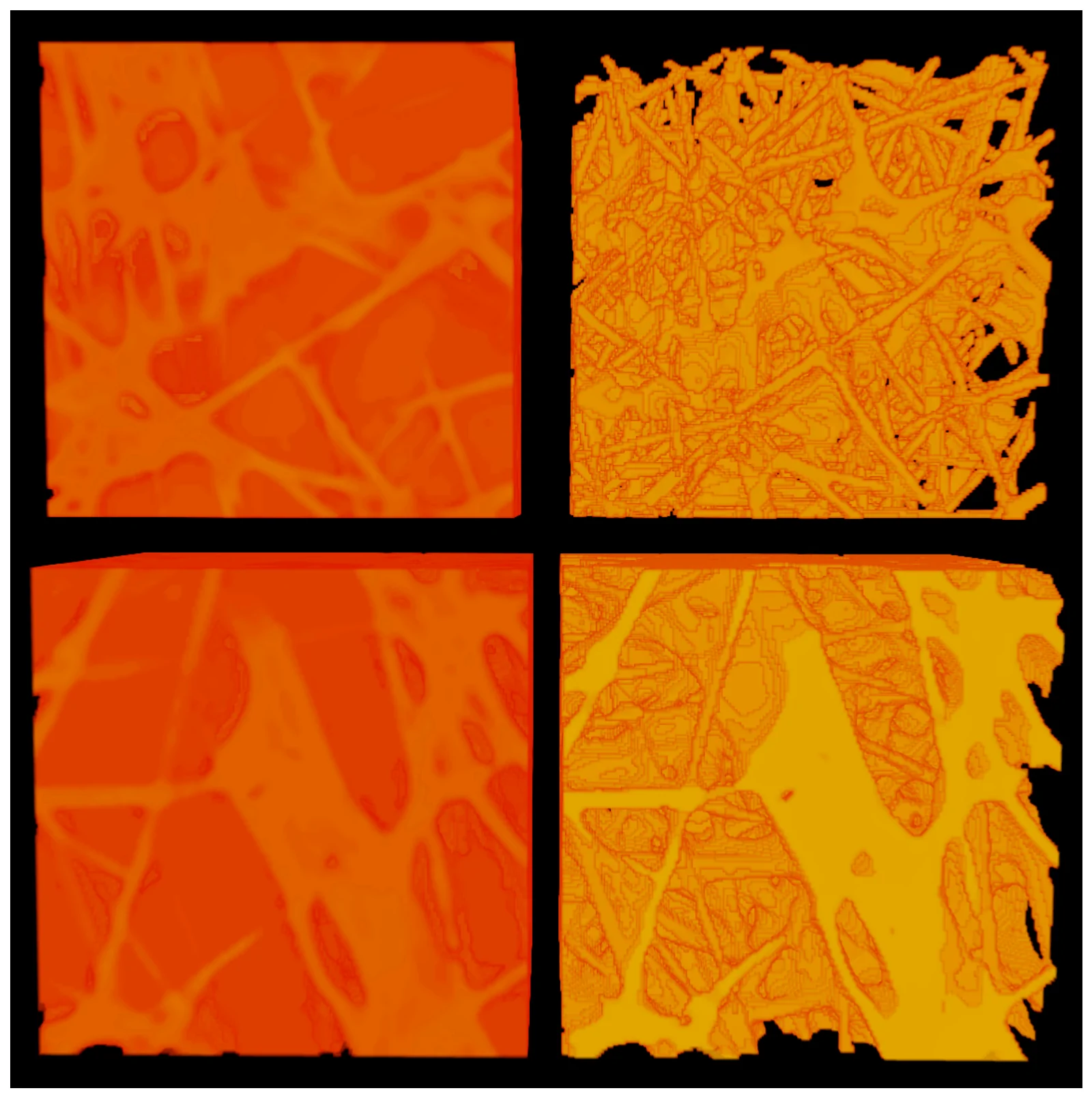
Prompt-driven comparison of two gas diffusion layer variants inside ASCRIBE-XR, generated from a single natural-language request. Rows correspond to GDLs coated by 5% (top, 5dry) and 60% (bottom, 60dry) PTFE; columns show, for each sample, the background-suppressed raw volume (left) and the fiber network isolated by masking (right). For each dataset the agent cropped an equal-sided central cube, subtracted the tenth-percentile intensity, applied a Yen threshold, removed connected components smaller than 500 voxels with scikit-image regionprops, and used the resulting mask to isolate the structure in the original cube; the four volumes were composited into the 2×2 layout and returned via submit_volume. All panels are directly manipulable in the shared immersive space.

Within ASCRIBE-XR, a researcher compared two manufacturing variants of the layer directly from a conversational request, without authoring any dataset-specific code. The prompt asked the agent to read the micro-CT stacks of two samples, here coated with 5% and 60% hydrophobic PTFE (the percentages are the mass ratio of the added PTFE to the untreated GDL), denoted 5dry and 60dry, to crop an equal-sided cube from the center of each, subtract the tenth-percentile intensity to form a background-suppressed “raw” volume, threshold that volume with the Yen method (taking the background as dark), remove connected components smaller than 500 voxels, and use the surviving mask to isolate the fiber network within the original cube. Each dataset, approximately 1 gigabyte each, was processed individually, and the four resulting volumes, raw and masked, for both samples, were composited into a single 2×2 arrangement and returned through the submit_volume tool. The agent performed the cropping, percentile subtraction, automatic thresholding, and scikit-image regionprops size filtering entirely within its server-side working directory. Figure 2 shows the composited result in the immersive space, where the denser fiber network and residual binder of the higher-PTFE sample can be contrasted directly against the lower-PTFE sample. Although this comparison required cropping, background subtraction, segmentation, and result arrangement across two datasets, the pipeline was executed using general-purpose image-processing libraries as well as a state-of-the-art vision transformer architecture via a single natural-language prompt. This use-case demonstrates how the LLM-based solution was able to use one prompt to orchestrate a complex, multi-dataset operation, highlighting how the agent complements the zero-shot, foundation-model pathway of Section 5.2 with classical, fully scriptable computer vision.

### 5.2. Use-Case II: Plant for Biofuel

*Panicum hallii*, a diploid model grass for bioenergy feedstocks such as switchgrass, was imaged at ALS beamline 8.3.2 (cropped microtomography with approximately 0.5 gigabytes) as a root specimen grown on agar. This use-case exercises the full prompt-driven, zero-shot segmentation pathway end to end: from a raw image stack on the server to a decimated surface mesh in the shared VR scene, with no dataset-specific code authored in advance.

Working from within the virtual environment, a researcher issued a single natural-language request: to load the volume from the multi-page TIFF stack on the server, subsample it by a factor of four, install and apply SAM [43] to isolate the plant structure and convert the segmentation to a surface, apply adaptive remeshing, and return the result. The agent satisfied this request entirely within its server-side working directory: it authored and executed Python to read the stack and decimate it, applied the promptable foundation model to isolate the root from the surrounding agar and background, requiring no task-specific training, extracted an isosurface of the resulting mask, and remeshed it adaptively to concentrate triangles where curvature was high before returning the lightweight geometry through the submit_mesh tool. The mesh was streamed into the scene and cached for the room, exactly as for the parametric generators described in Section 4.1.

Because the prompt named the model but not the manner of its use, every methodological choice that followed was made by the agent rather than by the researcher, and we report them here with details a reader might need to judge the results. The agent first searched the host for an existing checkpoint, found none, and installed the reference segment-anything package together with a CPU-only build of PyTorch, and it then retrieved the ViT-B checkpoint (sam_vit_b_01ec64.pth, 375 MB) from the model’s public distribution point. It elected the smallest of the three released backbones and ran inference on the CPU because no GPU was allocated to the server in this deployment. The volume was reduced from 1004×521×816 to 251×131×204 voxels by the requested factor of four in each axis.

SAM is a two-dimensional model, and the agent resolved the resulting dimensional mismatch by segmenting axial slices independently and reassembling them, processing every second slice, 126 of the 251, and interpolating between them afterwards. It used the promptable predictor rather than automatic mask generation, which meant it had to synthesize prompts without a user in the loop. Its heuristic was to treat local intensity variance as a proxy for the root: it computed a local standard-deviation map, retained the upper eighth percentile, took the largest connected component of that texture mask, and placed five positive point prompts near its centroid, together with four negative prompts at the image corners to suppress the surrounding medium. Of the three candidate masks SAM returned for each prompt set, it selected one by requiring that the mask cover between 2% and 50% of the frame and contain the seed point, and by then preferring the highest predicted-IoU score; the resulting per-slice scores ranged from 0.88 to 0.98. The stack of slice masks was cleaned by keeping the largest component per slice and filling holes, interpolated back to full depth, closed and hole-filled in three dimensions, and smoothed with a Gaussian kernel before marching cubes extracted the isosurface at the half-level. Isotropic explicit remeshing in PyMeshLab, run adaptively for five iterations at a target edge length of 1.5% of the bounding-box diagonal, reduced the raw surface from 214,708 vertices to 8778, a factor of roughly 24, which is what made the result cheap enough to stream into the headset. Slice-by-slice inference dominated the cost at approximately 1000 s on the CPU.

We present this pipeline strictly as an illustration of the agent’s autonomous scripting capabilities, rather than as an optimal or generalized segmentation methodology. The agent’s choice of independent per-slice processing lacks inherent three-dimensional coherence, and its variance-based heuristic encodes dataset-specific assumptions that may not transfer across domains. Furthermore, the mask-selection logic represents a functional engineering heuristic rather than a scientifically validated criterion. Ultimately, the significance of this demonstration lies in the platform’s ability to dynamically assemble and execute the pipeline, rather than in the scientific robustness of the agent’s specific algorithmic selections. This demonstration establishes that an external foundation model can be dynamically accessed and executed and its results rendered within the immersive scene via a single natural-language prompt. However, it does not constitute a quantitative validation of the resulting segmentation, an architectural distinction further elaborated in Section 7.

**Figure 3:**
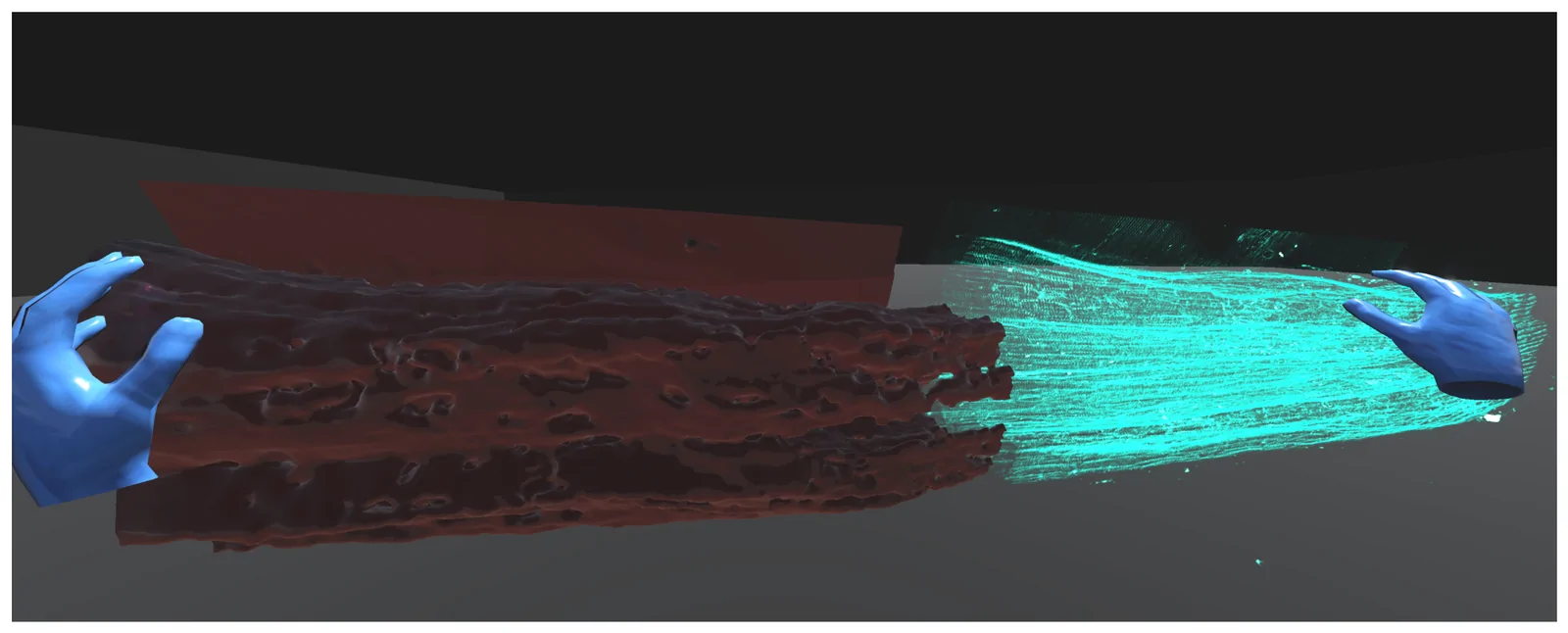
Prompt-driven zero-shot segmentation of a *Panicum hallii* root scan (ALS 8.3.2) inside ASCRIBE-XR. From a single natural-language request, the Claude agent loaded the TIFF stack, subsampled it, applied SAM to isolate the root structure, meshed and adaptively remeshed the segmentation, and returned the geometry via submit_mesh. Right: The raw volumetric rendering of the scan. Left: The resulting decimated surface mesh, streamed back into the shared scene. The two VR hands indicate that both representations are directly manipulable in the immersive space.

Figure 3 shows the outcome inside ASCRIBE-XR: the raw volumetric rendering of the scan on the right and the agent-generated surface mesh of the segmented root on the left, both manipulable in the same immersive space. Because the entire workflow was expressed as one prompt rather than a bespoke pipeline, the same interaction generalizes to other specimens in the catalog, the researcher changed the target named in the prompt, not the platform.

### 5.3. Use-Case III: High-Resilience Concrete

ASCRIBE-XR’s earliest demonstrations centered on ancient Roman concrete, whose crack-tolerant and self-healing behavior has made it a model system for the design of durable, high-resilience cementitious materials. Resolving the dense fragments and crack network within a micro-CT [48,49,50] volume (raw data with approximately 90 MB) of such a specimen, imaged at ALS beamline 8.3.2, again reduces to a single prompt.

**Figure 4:**
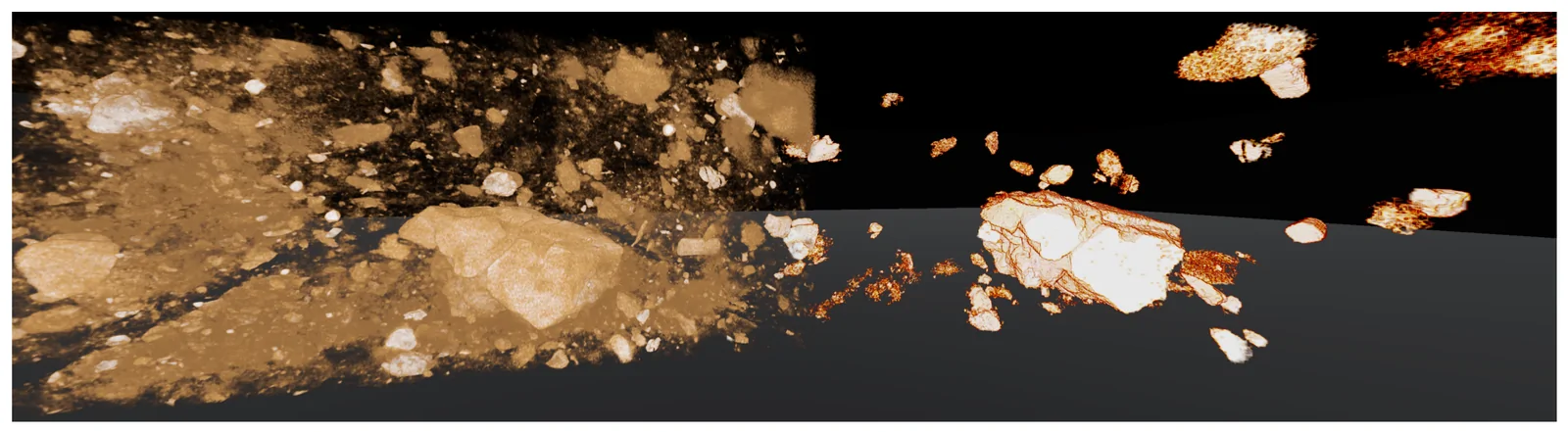
Prompt-driven volumetric segmentation of a high-resilience concrete micro-CT scan inside ASCRIBE-XR. The Claude agent subsampled the slice stack, thresholded each slice at intensity above 190, and removed connected components smaller than 1000 voxels using scikit-image regionprops, returning the cleaned field through submit_volume. The size-filtered high-density fragments (right) were rendered alongside the raw data (left) and remained manipulable as a volume in the immersive scene.

Here the researcher asked the agent to load the reconstructed slice stack, subsample it by a factor of two, threshold each slice at an intensity above 190, and remove connected components smaller than 1000 voxels before returning the cleaned volume. The agent carried out the thresholding and scikit-image regionprops size filtering within its server-side working directory and returned the result through the submit_volume tool rather than as a surface, preserving the full three-dimensional intensity field for direct volumetric inspection. Figure 4 shows the rendered volume in ASCRIBE-XR, with the size-filtered high-density fragments isolated against the surrounding matrix. Because the request again named only generic operations, such as threshold, size filter, and image transformation, the same interaction transferred to any volumetric specimen for which an intensity criterion was meaningful: the researcher edited the threshold named in the prompt, not the platform.

### 5.4. Performance Analyses

The choice of volume-rendering technique itself materially affects how the three-dimensional structure is perceived and interpreted [51], motivating a careful characterization of the renderer placed by ASCRIBE-XR in the path of every interactive session. To evaluate the performance characteristics of ASCRIBE-XR’s volumetric rendering pipeline, we benchmarked the raymarching renderer under varying dataset resolutions and raymarching step sizes. Performance overhead was measured by comparing baseline execution times against execution times with the raymarching shader enabled. For each configuration, GPU frame time and CPU processing time were recorded. The incremental cost introduced by the shader was computed as

(1) $$\Delta T=T_{\mathrm{shader}}-T_{\mathrm{baseline}},$$

where $T_{\mathrm{shader}}$ denotes the measured execution time with raymarching enabled and $T_{\mathrm{baseline}}$ represents the corresponding baseline measurement.

Tests and measurements were conducted as follows. Each configuration was run in a flat-screen desktop session rather than in a headset, at Godot’s default window size of 1152×648 pixels, using the Forward+ renderer with vertical synchronization disabled so that frame time was not clamped to a display refresh interval. Rendering to a desktop window rather than to a stereo headset target isolates the cost of the shader itself from the stereo rendering, reprojection, and compositor behavior that a runtime would otherwise contribute, and it makes the comparison across configurations depend only on the variable under study. Each configuration was allowed to run for 6000 frames of warm-up before any data were recorded, which covered shader compilation, texture upload, and mipmap generation, and the reported GPU and CPU times are then averages over the following 60,000 frames, drawn from Godot’s built-in per-frame rendering timers. All measurements were taken on a single workstation with a consumer NVIDIA RTX 3090 GPU, featuring 24 GB of GDDR6X memory and 10,496 CUDA cores.

These figures are intended to be illustrative rather than absolute. A single GPU, a single driver version, and a single window size cannot establish portable performance numbers, and the absolute microsecond values reported below would change substantially with different hardware, a different render target, or a stereo headset in the loop. What the measurements are meant to show is the shape of the cost function, that is, which parameters govern rendering cost and by how much they scale relative to one another, since it is that relative structure, and not the absolute frame time on our particular machine, that tells a user which control to reach for when a session must be brought within an interactive budget. Therefore, we report no variance across repeated runs and refrain from conclusions that depend on the absolute values.

**Figure 5:**
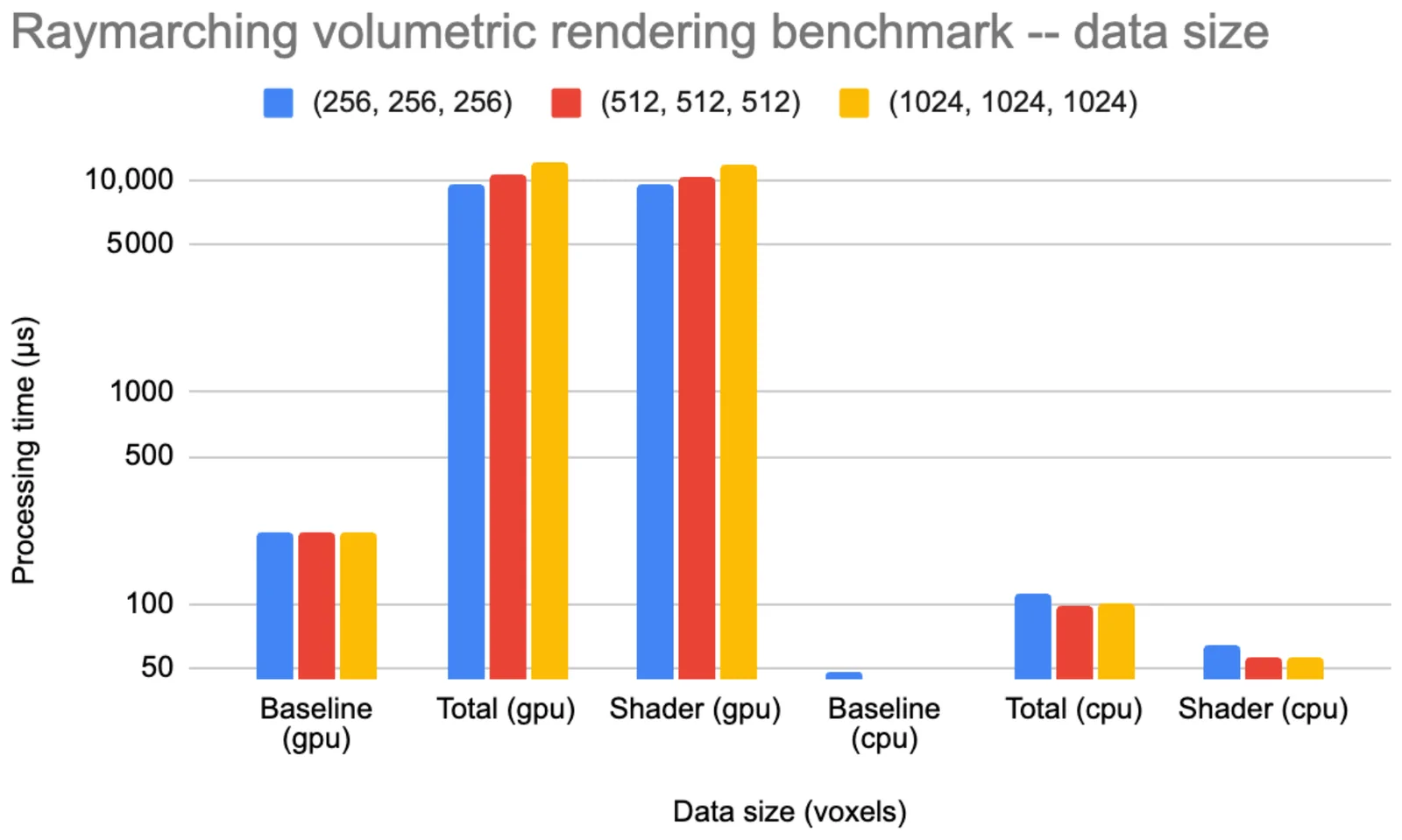
Effect of dataset resolution on raymarching performance. Processing time is reported in microseconds. GPU and CPU execution times for volumetric datasets ranging from $256^{3}$ to $1024^{3}$ voxels.

Figure 5 illustrates the impact of volumetric dataset size on rendering performance. Increasing the dataset resolution from $256^{3}$ to $1024^{3}$ voxels resulted in a GPU execution time increase from 9680 μs to 12,050 μs, corresponding to an overhead increase from 9460 μs to 11,830 μs per frame. In contrast, CPU execution times remained relatively stable, increasing only from approximately 100 μs to 110 μs. These results indicate that the shader rendering cost is dominated by screen-space ray count/step-size rather than texture size.

**Figure 6:**
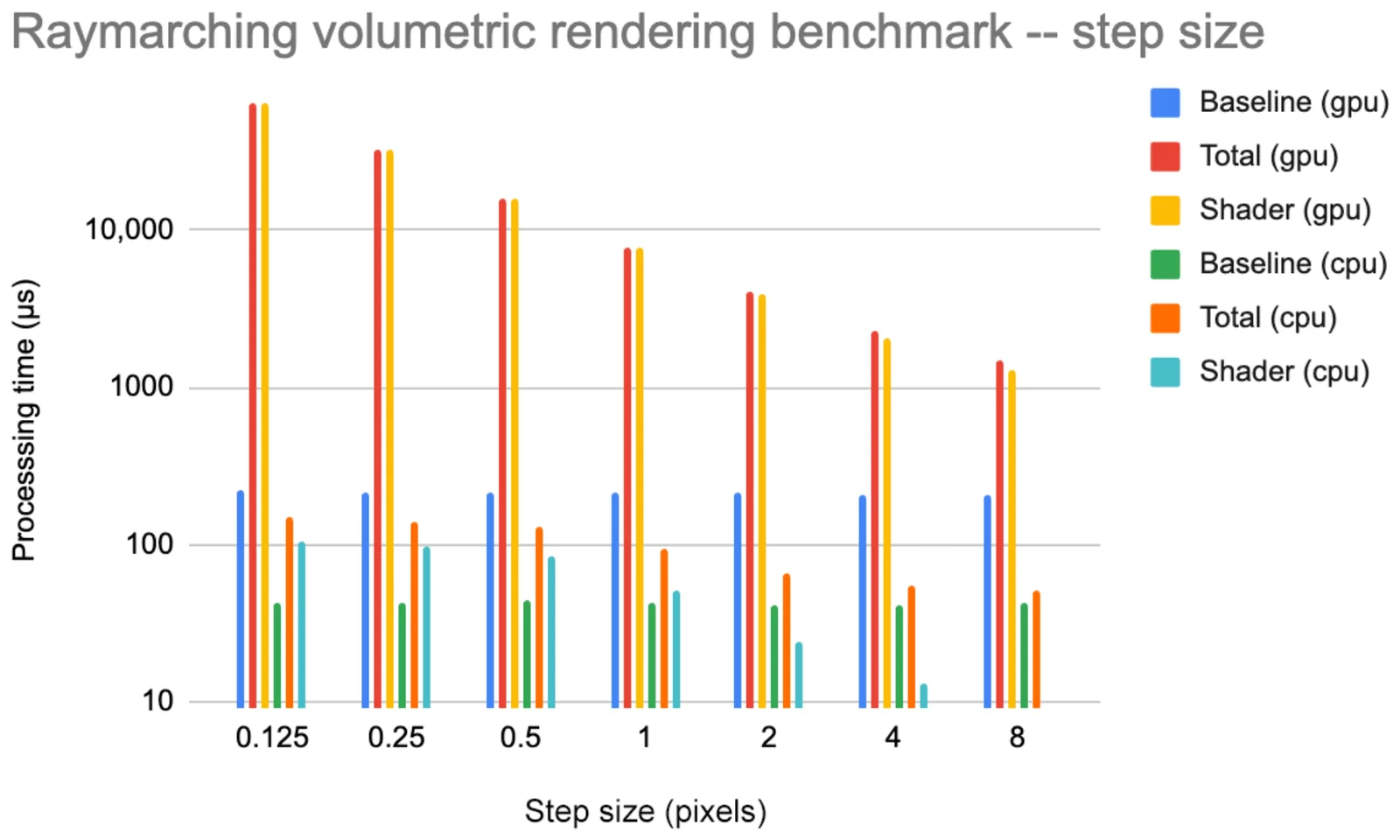
Effect of raymarching step size on rendering performance. Step size is expressed in pixels (the ray advance per sample relative to the screen), and processing time in microseconds. GPU and CPU execution times for raymarching step sizes ranging from 8 to 0.125 pixels. Smaller step sizes increase sampling density and therefore increase rendering cost. Benchmarks were performed on a consumer RTX 3090 GPU.

Figure 6 demonstrates the effect of raymarching step size on performance. As the step size decreased from 8 to 0.125 pixels, GPU execution time increased dramatically from 1500 μs to 64,220 μs, while the corresponding overhead increased from 1290 μs to 63,990 μs per frame. CPU execution time also increased from 51 μs to 149 μs, though its contribution remained small relative to GPU cost. This behavior is expected because smaller step sizes require substantially more sampling operations.

These results demonstrate that raymarching step size is the dominant factor governing rendering performance within ASCRIBE-XR. While larger volumetric datasets increase computational cost, the sampling density controlled by step size has a significantly greater impact on execution time. Expressed as ratios, which is the form in which these measurements are meant to be read, a 64-fold increase in voxel count raised GPU frame time by roughly a quarter, whereas a 64-fold increase in sampling density along each ray raised it by a factor of about 43. The practical implication is portability even though the underlying numbers seem differently: step size, not dataset resolution, is the control that governs whether a session renders at an interactive rate.

Beyond rendering, we characterized the end-to-end latency of the prompt-driven generation workflow itself. Table 2 decomposes a representative “AI Generate” request—from the moment the user submits the prompt in the headset to the moment the resulting specimen is displayed—into its constituent stages across ASCRIBE-XR, ASCRIBE-Link, and the hosted agent provider. This is a single instrumented run, reported as an illustrative decomposition of where time is spent rather than as a benchmark. We did not repeat it and therefore report no variability; agent inference time in particular depends on the prompt, on the analysis the model chooses to perform, and on the load and version of a hosted service outside our control, so a distribution gathered on one day would not transfer to another. The measurement supports one observation only, namely that in this run the stages implemented by our system were together small besides the agent’s inference time.

## 6. Availability and Deployment

ASCRIBE-XR is distributed as open-source software for local deployment. There is no hosted service, and the platform is not listed in a headset application store; both components are obtained from public Git repositories. To lower the barrier for users who wish to try the client rather than build it, we additionally published signed Android packages for standalone headsets with the client’s tagged releases, so that evaluating the viewer requires only sideloading a package, while the server that makes the analytical features work, is installed from source. The VR client is released under a modified BSD license (https://github.com/lbl-camera/Ascribe-XR, (accessed on 1 August 2026)), and the ASCRIBE-Link server is available at https://github.com/ronpandolfi/Ascribe-Link, (accessed on 1 August 2026). This subsection summarizes what a user needs in order to run the platform; the intent is that a single workstation with a Meta Quest 3/3S headset is sufficient to reproduce everything reported here, and that the collaborative and agentic features are additive rather than prerequisite.

### 6.1. Client: Installation and Supported Devices

The client is a Godot 4.6 project using the Forward+ renderer. A released Android package can be installed directly on a standalone headset with adb or a sideloading tool. To build instead from source, the Godot editor and the matching export templates are required; two export targets are configured, a Windows x86-64 desktop build and an Android build for standalone headsets, and producing the latter additionally requires the Android SDK, a JDK, and a signing keystore. Interaction is through OpenXR, so the desktop build runs against any conformant runtime, including SteamVR, Meta Link, and Virtual Desktop, and this is the configuration we recommend for the large volumes typical of synchrotron imaging, since the volume is rendered by the workstation GPU. The standalone Android target is configured for Meta Quest 3; Meta passthrough is enabled through the vendor plugin, while hand, eye, face, and body tracking are disabled, so interaction is controller-based on all devices. Other OpenXR headsets are expected to work through PC-VR but have not been tested. For development, review, and demonstration without a headset, the project autoloads an XR simulator that drives the scene from keyboard and mouse, so the client can be run and inspected on an ordinary desktop.

We do not publish a hard minimum specification, as the binding constraint is the dataset rather than the application. The client uploads each specimen to the GPU as a 3D texture, so video memory must accommodate the volume at its streamed resolution, and the raymarching cost reported in Section 5.4 scales with step size and viewport rather than with headset model. The benchmarks in this paper were collected on a consumer RTX 3090; step size is exposed as a continuous control precisely so that a session can be brought within an interactive frame budget on less capable hardware without altering the volume on the server.

### 6.2. Server: Installation and Network Requirements

ASCRIBE-Link requires Python 3.11 or later and it can be installed from a clone of the repository with pip install -e., which provides the ascribe-link console script. Its runtime dependencies are NumPy, PyVista, Pillow, websockets, and the Litestar ASGI framework; the service is deployed via Uvicorn and binds 0.0.0.0:8000 by default, with the specimen directory, host, and port settable from the command line. An OpenAPI description of the REST interface is served at /docs. The client is pointed at the server through a single configuration value, which defaults to http://127.0.0.1:8000 for a workstation running both components; a remote or standalone headset instead needs the server’s address on a reachable network. The optional federation mode adds an outbound WebSocket connection from each worker to a relay, which is how a compute node behind an institutional firewall can contribute specimens to a session without accepting inbound connections.

The server currently performs no authentication, terminates no TLS, and accepts cross-origin requests from any origin. It is therefore intended to run on a trusted laboratory network, or behind a reverse proxy or VPN that supplies those controls, and should not be exposed directly to the public internet. We state this explicitly because the agent layer described below executes code on the host.

Multi-user sessions require, in addition, reachability between peers. Connection state is exchanged over MQTT carried on WebSockets, for which the distribution points at a laboratory-hosted broker by default, and the media and data channels are then established directly between peers over WebRTC, with voice carried on the same sessions using Opus. For internet connections, peer discovery defaults to a public STUN server. Because restrictive network environments (such as symmetric NAT) may block these direct connections, the client allows users to supply a fallback TURN service via an optional ICE configuration file. Additionally, for co-located researchers sharing a local network, the platform provides a direct ENet transport that bypasses these external routing requirements entirely.

### 6.3. Agent Layer and Model Access

The agent layer is optional and off by default; it is enabled with the --enable-agent flag and requires the agent extra, which installs the Claude Agent SDK. Everything else, including the REST interface, the Specimen Catalog, volumetric and mesh rendering, and multi-user sessions, functions without the agentic layer. The SDK operates by spawning the Claude command-line client as a subprocess, so the client, and the Node.js runtime it requires, must be installed and already authenticated on the server host. ASCRIBE-Link does not handle credentials itself and stores no key: access to the model is whatever the Claude client has been configured with, which may be an Anthropic API key or a subscription login. The use of the agent therefore entails a dependency on a commercial service and its associated cost, which is the principal caveat on the platform’s otherwise self-contained deployment. The model is selected with the --agent-model flag, whose default at the time of writing is claude-opus-4-8; the exact SDK and package versions used for the results reported here are listed in the Supplementary Material.

When deploying ASCRIBE-XR with the agent layer enabled, users must account for two critical architectural considerations regarding execution security and model dependencies.

First, because the agent authors and executes arbitrary code on the server host, execution isolation is paramount. On Linux deployments, ASCRIBE-Link routes execution through a Firejail sandbox. This profile enforces a private working directory, applies seccomp filters, drops root capabilities, disables network access during execution, and imposes strict CPU and memory limits to prevent resource exhaustion. However, we explicitly note that this configuration serves as a practical safeguard against malformed or runaway scripts rather than an audited security boundary against adversarial prompt injection. If Firejail is absent—or if the server runs on Windows, as was the case for the technical demonstrations in Section 5—execution falls back to an unconfined state with a logged warning. Consequently, production deployments should isolate the server host within a dedicated virtual machine or container with strictly partitioned data access.

Second, ASCRIBE-Link does not bundle internal analysis models or foundation weights. Instead, the agent dynamically scripts access to external libraries and weights at runtime within its designated working directory, as demonstrated by the segmentation pipeline in Section 5.2. While this dynamic dependency injection allows the platform to utilize tools it was never explicitly programmed to support, it inherently ties reproducibility to the host environment. Therefore, replicating these results depends entirely on the exact package environment, SDK versions, and system prompts documented in the Supplementary Material, rather than on the base installation of the platform alone.

### 6.4. Software Versions

Because the analysis in an agent-driven session is produced at run time rather than fixed in the source code, the identity of the model and of the surrounding software environment is part of the non-deterministic algorithm rather than an implementation detail. Therefore, Table 3 records the versions in use for the demonstrations reported in this paper.

Notice that the model is identified by the provider’s version string, claude-opus-4-8, which is the level of specificity a hosted service exposes; we cannot pin model weights the way a package version is pinned, and results obtained through a hosted model are consequently reproducible only in the weaker sense that the same request, addressed to the same advertised model version, should yield a comparable analysis rather than an identical one. The agent layer has two version numbers rather than one because the Python claude-agent-sdk does not itself contact the service; it spawns the Claude Code command-line client, whose version is recorded separately, together with the Node.js runtime that client requires.

The segmentation entries are deliberately distinguished from the rest of the table. Because the platform ships no analysis models, the semantic segmentation based on a foundation model described in Section 5.2 used a package and checkpoint the agent obtained at run time inside its working directory, which is discarded when the job completes and is not part of the server environment. Henceforth, those entries are recovered from the archived session logs described next, rather than read from the installed software. That asymmetry is the practical form of the provenance issue this type of automated system creates: the part of the method that varies most is the part the installed environment does not record.

### 6.5. Archived Agent Sessions

We archived each demonstration as a self-contained record; the three corresponding files are provided as Supplementary Material and are also published in the server repository under example-output/, so that they remain alongside the software that produced them and can be updated as it evolves. Each contains the verbatim prompt, a full transcript of the session including every tool call and its result, the Python code that the agent wrote, the console output of each stage, and the array or mesh that was submitted to the client. For the segmentation case the archive additionally records the package installation, the checkpoint download with its source URL and byte size, the intermediate mask stacks, and diagnostic renderings of the segmentation. The intent is that the numbers quoted in Section 5.2, and the choices attributed there to the agent, can be checked against the record rather than taken on our word, and that a reader may re-execute the analysis exactly as it was performed.

There are two considerations about reproducibility using these archives. First, they were produced by re-executing the three prompts under the environment described in Table 3, and are therefore representative of the workflows the figures depict rather than being the identical sessions that produced them; the figures come from earlier runs of the same prompts. Both the figures and the archived sessions were, in our judgment as the researchers who ran them, fair representatives of the usual result rather than selected best cases. Second, the re-executions did not reproduce their predecessors step by step. The analytical approach and the character of the result were consistent across runs, but specific choices left open by the prompt, including thresholds, intermediate parameters, and the structure of the generated code, differed.

The observed execution variability is a deliberate consequence of prompt design, not an architectural constraint. The prompts utilized in our demonstrations are intentionally under-specified—naming a high-level goal (e.g., “run SAM segmentation to isolate the plant structure”) without dictating hyperparameters, cross-section processing methods, or mask selection criteria. This under-specification simulates an exploratory workflow, proving the system’s capacity to successfully generate analytical pipelines without pre-existing, dataset-specific code. While this open-ended adaptability inherently introduces variance, prompt specificity functions as a precise control mechanism. A highly specified prompt that fixes exact thresholds, sampling rates, and meshing parameters tightly constrains the agent, yielding the repeatable outputs required of a mature protocol. Furthermore, because the agent dynamically generates standard code, the archived script from any run can be extracted and executed directly, rendering the analysis fully deterministic. Ultimately, the architecture supports the entire discovery lifecycle, allowing researchers to leverage exploratory flexibility initially and enforce strict reproducibility as an analysis matures.

While prompt specificity provides the architectural mechanism for controlling variability, statistically quantifying this behavior falls outside the scope of this systems demonstration. The illustrative executions presented here are designed to prove platform integration rather than to establish a baseline success rate, define a comprehensive error taxonomy, or quantify outcome divergence. We identify the systematic characterization of agent variability, here evaluated across extensive iterations and varying levels of prompt constraints, as a critical avenue for future empirical work.

## 7. Discussion

A primary limitation of existing immersive scientific tools is their detachment from the underlying computational workflows that generate the data. To address this, we integrated a dynamic interface, a customizable catalog, and an agent-assisted segmentation layer into the ASCRIBE-XR architecture. Taken together, these components demonstrate how an immersive viewer can be expanded beyond an output display into an interactive computational workspace.

In its pre-release, ASCRIBE-XR presented a fixed menu of specimens whose analysis had already been completed in external pipelines; the immersive environment is mainly a place to audit results, and not designed for segmentation itself. The Python REST interface, the metadata-driven Specimen Catalog, and the Claude Agent SDK layer together move that analytical boundary inside the shared space. A single natural-language request drives essential, fully scriptable computer-vision pipelines for the fuel-cell gas diffusion layers (Section 5.1), a zero-shot foundation-model segmentation for the *Panicum hallii* root (Section 5.2), and a volumetric intensity segmentation for high-resilience concrete (Section 5.3). These are three domain sciences and two distinct analytical paradigms, none of which required dataset-specific client code. This is the multi-domain generalization argued for in Section 4.1 demonstrated in practice rather than asserted: adapting to a new problem is a matter of what the researcher says, not of what the platform is rebuilt to do.

It is important to note that these prompts are not natural-language scripts that simply rename the steps of a fixed pipeline; they delegate genuine analytical decisions to the agent. A researcher can pose an exploratory request as loosely as “I’m curious how this looks with zero-shot SAM: show me an example,” and the agent resolves the unstated choices (how to apply the model, at what resolution, with what meshing) on its own, making the interaction closer to autonomous agentic analysis than to scripted automation.

The architecture also matters for who can participate. Keeping full-resolution datasets resident on remote compute servers and streaming only the reduced result, whether a decimated mesh or a subsampled volume, addresses the standing tension between the fidelity scientists need and the limits of consumer-grade XR hardware, an arrangement we expect to help keep interaction latency within comfortable bounds, though we have not measured that directly. Performance analysis quantifies the client-side cost of that arrangement. Rendering is firmly GPU-bound while CPU contributions remained on the order of $10^{2}\;\mu$s across every configuration, and dataset resolution is not the dominant factor: a fourfold increase in linear resolution, from $256^{3}$ to $1024^{3}$ voxels, corresponding to a 64-fold increase in voxel count, raised GPU frame time by only about a quarter. Raymarching step size, by contrast, spanned nearly two orders of magnitude in cost over the tested range. This is a practical result rather than a limitation: step size is a single, continuously adjustable control that lets a session trade sampling density against frame rate to stay within an interactive budget on whatever hardware is at hand, while the underlying volume remains untouched on the server.

The implications of these capabilities must be contextualized within the architectural scope of this study. Generating analysis on demand rather than fixing it in advance raises reproducibility and provenance questions that the present implementation does not settle: the fidelity of a result depends on the model and on the prompt that elicited it, and the code that produced it is not yet retained as a durable artifact. It also means that model-authored code executes on the server host. As described in Section 6, the Firejail wrapper we apply on Linux is a practical guard rather than an audited security boundary, and we regard proper isolation of the execution environment as an open engineering problem for this class of system rather than as a solved aspect of ours. Mesh decimation, which is what makes streaming to the headset feasible, necessarily discards geometric detail, and the appropriate degree of that trade-off is domain-specific. The present integration is deliberately narrow; it presents a single agent exposing three result-returning tools, and the benchmarks characterize the renderer on a single RTX 3090 GPU rather than the end-to-end latency of a live collaborative session. We regard these as the natural boundaries of a first service-oriented release rather than obstacles to it.

These architectural boundaries also define a clear trajectory for future development. Capturing the code the agent authors as a durable, inspectable record would turn each conversational analysis into a reproducible artifact; broadening the tool surface and moving beyond a single agent would let more elaborate workflows be expressed in the same conversational idiom; and controlled user studies would establish whether the immersive, prompt-driven setting measurably improves comprehension and expert-to-novice knowledge transfer over conventional tools, a claim the architecture is designed to support but that remains to be tested directly.

## 8. Conclusions

The primary objective of ASCRIBE-XR is to unify immersive scientific visualization with the active computational auditing of complex segmentation results. By evolving from a monolithic VR viewer into a modular, service-oriented platform, the system addresses the inherent rigidity of traditional XR tools. The integration of a Python REST/JSON interface, a domain-invariant Specimen Catalog, and conversational, single-agent pipelines orchestrated by the Claude Agent SDK creates a highly adaptive environment. This architecture facilitates collaborative, real-time exploration of massive datasets while providing an ecosystem capable of adapting to diverse analytical needs across scientific, industrial, and educational domains.

The evidence we offer for this capability is strictly architectural: our use-cases serve as technical demonstrations that a single natural-language request can drive segmentation and meshing across unrelated fields without requiring dataset-specific client code. Moving forward, the platform’s development will focus on two parallel trajectories to transition this architecture into a more reproducible scientific toolset. Computationally, future work must address the open engineering challenges of execution security and data provenance by hardening sandbox isolation and capturing dynamically generated analysis code as durable artifacts; establishing this secure foundation will subsequently enable the expansion to more elaborate multi-agent workflows. Human-centrically, the architecture serves as the necessary apparatus to empirically test core hypotheses of immersive analytics. Specifically, controlled user studies against conventional desktop tools are the required next step to quantify whether this prompt-driven, shared scene measurably improves spatial comprehension, eases expert-to-novice knowledge transfer, and mitigates discomfort through low-latency streaming of higher resolution volumes and geometries.

## Figures and Tables

**Table 2:** Illustrative end-to-end timing of one “AI Generate” request (a single instrumented run producing a volumetric result in a 69.7 MB binary envelope; not repeated, so no variability is reported). In this run, agent inference—the time the hosted model provider spent reasoning and writing generation code—accounted for ∼136 s, one to two orders of magnitude above any single stage implemented by our system; all ASCRIBE-XR and ASCRIBE-Link stages combined (submission, agent startup, result capture, transport, and client-side construction) accounted for ∼12 s, of which network transport of the ∼70–90 MB payloads was the largest share. The decomposition is presented to show where time is spent in the pipeline, and not as a performance benchmark: inference time varies with the prompt, with the analysis the model elects to perform, and with the load and version of a service outside our control.

Stage	Location	Time (s)
Prompt submission (client → server)	Network	0.01
Agent startup (job dispatch, CLI spawn, first message)	ASCRIBE-Link	2.0
Agent generation (query → submission tool)	Agent provider	136.4
Result submission tool (load, validate, encode)	ASCRIBE-Link	0.4
Agent teardown and job finalization	ASCRIBE-Link	5.1
Result transport (poll, fetch, envelope download)	Network	4.8
Specimen construction and display	ASCRIBE-XR	0.06
**Total (submit → displayed)**		**148.5**

**Table 3:** Software environment used for the agent-driven demonstrations reported in Section 5. The segmentation entries are separated because that stack is not part of the installed platform: the agent installed it at run time in response to the prompt, and it is recorded here from the archived session (Section 6.5) rather than from the server environment. The complete environment specification is provided as Supplementary Material.

Layer	Component	Version
Client	Godot engine (Forward+ renderer)	4.6
Server	Python	3.12.0
	ascribe_link	0.2.0
	Litestar/Uvicorn	2.21.1/0.42.0
	websockets	16.0
Agent	Language model	claude-opus-4-8
	claude-agent-sdk (Python)	0.1.50
	Claude Code CLI (spawned by the SDK)	2.1.81
	Node.js runtime	24.7.0
	mcp (Model Context Protocol)	1.26.0
Analysis	NumPy/SciPy	1.26.4/1.12.0
	scikit-image	0.22.0
	tifffile	2025.6.11
	PyVista/VTK	0.45.3/9.4.2
	PyMeshLab/Trimesh	2025.7.post1/4.7.1
	Pillow	11.3.0
Segmentation	segment-anything	1.0
(agent-installed	SAM backbone/checkpoint	ViT-B, sam_vit_b_01ec64.pth
at run time)	PyTorch/TorchVision (CPU build)	2.13.0/0.28.0

## Data Availability

Code supporting reported results is available at https://github.com/lbl-camera/Ascribe-XR (VR client, accessed on 1 August 2026), and https://github.com/ronpandolfi/Ascribe-Link (server, accessed on 1 August 2026); deployment requirements for both are summarized in Section 6. Data is available as in the article. Demo and other requests can be made by contacting the authors at ronpandolfi@lbl.gov and dushizima@lbl.gov. New releases and updates may also be available at https://sites.google.com/lbl.gov/ascribe (accessed on 1 August 2026).

## Supplementary Materials

The following supporting information can be downloaded at https://www.mdpi.com/article/10.3390/jimaging12080393/s1. Three archives of agent sessions, one per use-case, are provided as described in Section 6.5: S1, membrane-agent-artifacts (fuel-cell gas diffusion layers; inputs 5dry.tif and 60dry.tif); S2, plant-agent-artifacts (*Panicum hallii* root; input rec20201028_190153_wet2_pipette_z50_YESagar_x00y01.tif); and S3, concrete-agent-artifacts (high-resilience concrete; stack LOAD5_rec20220824_c3_comp_05_y0002 _verticalcrop, 200 slices). Each archive contains the verbatim prompt, the complete session transcript including every tool call and result, the Python authored by the agent, the console output of each stage, and the submitted array or mesh. The segmentation archive additionally records the package installation and the checkpoint download; the checkpoint itself is not redistributed and is retrieved from the URL recorded in the log. Such logs are published in the ASCRIBE-Link repository under example-output/ (https://github.com/ronpandolfi/Ascribe-Link/tree/master/example-output, accessed on 1 August 2026); S4: ASCRIBE-XR VR Workspace.

## Abbreviations

The following abbreviations are used in this manuscript:

AI	Artificial Intelligence
BSD	Berkeley Software Distribution
CAD	Computer Aided Design
ICE	Interactive Connectivity Establishment
JDK	Java Development Kit
MCP	Model Context Protocol
MicroCT	Micro-Computed Tomography
MQTT	Message Queuing Telemetry Transport
NAT	Network Address Translation
PTFE	Polytetrafluoroethylene
SAM	Segment Anything Model
SDK	Software Development Kit
STUN	Session Traversal Utilities for NAT
TURN	Traversal Using Relays around NAT
VR	Virtual Reality
WebRTC	Web Real-Time Communication
XR	Extended Reality

## Appendix A

**Table A1:** Godot plugins used in ASCRIBE-XR.

Component Name	Primary Function	Repository Link
godot-xr-tools	Provides foundational scenes and scripts for AR/VR interaction and functionality.	https://github.com/GodotVR/godot-xr-tools/ (accessed on 1 August 2026)
godot_openxr_vendors	Enables support for vendor-specific OpenXR extensions and features (e.g., for Meta, Pico).	https://github.com/GodotVR/godot_openxr_vendors/ (accessed on 1 August 2026)
twovoip	Implements high-quality voice communication using Opus compression and denoising.	https://github.com/goatchurchprime/two-voip-godot-4 (accessed on 1 August 2026)
xr-autohandtracker	Enables gesture-based controller simulation using OpenXR hand tracking data.	https://github.com/Godot-Dojo/Godot-XR-AH (accessed on 1 August 2026)
xr-simulator	Allows for desktop-based simulation and testing of XR applications without a headset.	https://github.com/Cafezinhu/godot-vr-simulator (accessed on 1 August 2026)
webrtc-native	Official Godot plugin for peer-to-peer WebRTC communication in native applications.	https://github.com/godotengine/webrtc-native (accessed on 1 August 2026)
godot-mqtt	Implements the MQTT client protocol for lightweight messaging and signaling.	https://github.com/goatchurchprime/godot-mqtt/ (accessed on 1 August 2026)
player-networking	Custom high-level multiplayer logic, modified from a base demo.	https://github.com/goatchurchprime/godot_multiplayer_networking_workbench (accessed on 1 August 2026)
Terrain3D	A high-performance GDExtension for creating and rendering large-scale terrains.	https://github.com/TokisanGames/Terrain3D/ (accessed on 1 August 2026)
stl_importer	Imports STL (stereolithography) files from CAD and 3D printing applications.	https://github.com/grabthefish/StlImporter (accessed on 1 August 2026)
volume_layered_shader	A specialized shader and addon for rendering 3D volumetric data (e.g., MRI scans).	https://github.com/blackears/godot_volume_layers (accessed on 1 August 2026)
gdUnit4	Unit testing framework for Godot scripts, used for the project’s automated test suite.	https://github.com/MikeSchulze/gdUnit4 (accessed on 1 August 2026)

**Table A2:** Verbatim natural-language prompts issued to the ASCRIBE-XR agent for each use-case, with notes on aspects left unspecified—details the agent resolved autonomously rather than being directed.

Use-Case	Prompt (Verbatim)	Open-Ended Aspects (Agent-Resolved)
Plant root (*Panicum hallii*)	“Load the plant volume from tif stack at ^~^\Downloads\rec20201028_190153_wet2_pipette_z50_YESagar_x00y01_8bitcrop-roi.tif. Subsample it by a factor of 4. Run SAM segmentation to isolate the plant structure and generate a mesh. Then run adaptive remeshing. Submit the final result mesh.”	SAM was requested but no directions on how to apply it—the agent chose to run it slice-by-slice rather than volumetrically. The prompt for the “plant structure” gave no seed points, prompts, or class labels, leaving target selection to the agent. Subsampling axes/method, meshing algorithm (e.g., marching cubes) and isovalue, and the remeshing target resolution and quality criteria were all unspecified.
Roman concrete	“Load the Concrete data volume from the tif slices in reconstructedromanconcrete.tif, downsample by a factor of 2, then threshold each slice at t>190, then use skimage.regionprops to filter out objects smaller than 1000 voxels. Submit the resulting volume data.”	“Objects” and connectivity for labeling were undefined—whether components are found per-slice (2D) or across the volume (3D), and the neighborhood used, were left to the agent. The threshold direction (>190 retained as foreground), the downsampling method (subsample vs. interpolate) and axes, and the output dtype/format of the “resulting volume” were unspecified.
Wet/dry comparison (5 dry, 60 dry)	“Read the tif stacks at ^~^/Downloads/5dry.tif and ^~^/Downloads/60dry.tif and slice out a cube of equal length/width/height from the center. Perform threshold using Yen method from skimage, assuming the background is dark. Then use skimage.regionprops to filter out objects smaller than 500 voxels. Use that result to mask the original ‘cube’ data. Return a 2 × 2 stack of the raw and masked data. Each dataset should be processed individually.”	The cube edge length was not given—the agent chose the largest cube fitting each (possibly differently sized) volume. Whether Yen is computed per-slice or on the whole cube, and 2D vs. 3D connectivity for small-object removal, were unspecified. “Mask the original data” left the fill value for removed regions open. The layout of the “2 × 2 stack” (arrangement, axis order, spacing between the four volumes) was not defined.
